# An insight into patients’ perspectives on barriers affecting participation in shared decision making among patients with diabetes mellitus in Malawi

**DOI:** 10.1186/s12875-022-01635-9

**Published:** 2022-03-10

**Authors:** Martha Makwero, Adamson S. Muula, Felix Chima Anyanwu, Jude Igumbor

**Affiliations:** 1 Department of Public Health, University of Malawi, Kamuzu university of health sciences, Blantyre, 3 Malawi; 2 School of Public Health, Faculty of Health Sciences, University of the Witwatersrand, Johannesburg, South Africa

**Keywords:** Diabetes mellitus, Patient participation, Patient involvement, Shared decision making

## Abstract

**Background:**

Patient participation in decision making is a basic tenet for a patient centred care experience and, has potential to improve care experiences and responsiveness in chronic diseases such as Diabetes Mellitus (DM). However, documented experiences show that patient participation in decisions making is wanting. As Malawi strives to institutionalise patient centred care delivery, it is important to examine patients’ experiences and perceptions to identify barriers affecting their participation in shared decision making because this may provide evidence supporting strategies in implementation of the institutionalisation.

**Aim:**

The study sought to describe perspectives about barriers to participation in shared decision making among patients with DM in Malawi.

**Methods:**

This was an exploratory qualitative study. We targeted patients attending DM clinics in four public health facilities in southern Malawi from September to December 2019. We used In-Depth Interviews and Focus Group Discussions. Data was managed using Nvivo version 11 software and analysed using Content Analysis.

**Results:**

The study highlights the values, perceptions and benefits of shared decision making. Furthermore, patients’ narratives expose the struggles and vulnerabilities in their attempts to engage their providers towards shared decision making.

**Conclusion:**

Interactional power imbalances, insufficient dialogue and patients’ own restrictive attitudes towards engagement with their providers thwarts SDM in clinical encounters. To make SDM a reality, transforming medical education that emphasizes on the value of good patient-provider relationship and providers’ attitudes to regard patients as active partners may be a good starting point. Additionally, strategies that empower and change patients’ perceptions about SDM require investment.

**Supplementary Information:**

The online version contains supplementary material available at 10.1186/s12875-022-01635-9.

## Background

Investing individual or collective patients’ participation in shared decision making (SDM) is a fundamental step in enhancing patient centred care (PCC) experience because it enables patients to be active recipients of their care [1–3]. In practice, however, patients’ experiences of participation in medical decisions has been reported to lack deserved consideration and, often is limited to informing patients about providers’ care plans and expecting them to comply with them [4–6]. In Malawi, insufficient communication between patients and providers and, patients’ passivity around their care decisions are reported to impede PCC and its resultant benefits [7]. Yet, there is limited understanding of barriers that impede engagement in the SDM process in Malawi [7, 8].

Patient participation in shared decision making refers to interactional elements between a patient and a provider where a patient provides information, asks questions and shares experiences that contribute to collaborative problem exploration, identification of management options in a conducive space for dialogue [9, 10]. Optimal participation therefore facilitates the provision of care that meets at the nexus of patient preferences, provider experiential practice, available resources and evidence thereby potentiating the patient-provider dyad to reach patients’ personalised goals with the provider as a resource [1, 11]. Particulary in patients with Diabetes Mellitus (DM), participation in decision making enhances positive patient’ experiences of care, treatment goal setting, medication adherence, safety, glycaemic control and lifestyle modification through enhancement of patient self-efficacy [1, 12, 13]. Therefore, patient participation in SDM is a key enhancement in DM care as patients have to constantly make decisions to accommodate their complex individual situations, recurrent hospital visits, the disease, its treatment and goals [11].

Castro et al. identifies key dynamic processes in the patient-provider interaction as key antecedents to patient participation in SDM [10]. The processes include deliberate effort to solicit a patient or their proxies to participate in a supportive dialogue; engaging the patients in meaningful dialogue about the disease and the individualised treatment options available; and providing a supportive and safe interactional environment aimed at activating and empowering a patient to actively participate to their desired extent, refraining from merely validating providers’ agenda [10]. The process further requires acknowledgement of a patient as an expert of their disease [14]. The highlighted processes engender a well informed and activated team of patients and providers that is better placed to meet the demands of DM care [6, 15].

Despite the documented positive gains from patient participation, the practice in SDM in DM care is marked with failings [4, 7, 11, 16]. An interplay of patient, provider, system and contextual factors has been reported as affecting individualised levels of participation in SDM [10, 17]. At the patient-provider interactional level, factors known to influence DM care dynamics include patient’s disease and socio-economic profiles, patients’ educational status, health literacy, past experiences, patients’ views and cultural values [18]. For example, a patient’s low socio-economic status and socio-cultural backgrounds with centralized repressive government power that excludes potential challengers are reported to have lowered the likelihood of initiating engagement with a medical authority [12, 16], This element has been observed to create variability in observed patterns of SDM among different populations. Similarly, providers’ communication skills, attitudes towards shared decision making and conflicting roles are known to affect observed levels of patient participation [9, 10, 16]. Shortus et al. report that providers often are ambivalent about the extent which they can engage their patients towards personalised versus protocol-based biomedical goals [9]. Often, their ambivalence leads to non-solicitation of SDM around goals of care and expectation of trust from their patients [19]. At institutional level, interactional time constraints, and at systems level, contextual issues such as an authoritarian socio-political context affect participation in SDM [16].

The attention given to user active participation in SDM is increasingly becoming fundamental to quality service delivery discourses in Malawi’s chronic care settings [7, 20]. As Malawi endeavours to achieve greater active individual or collective participation especially in chronic patients, this paper uniquely presents patients’ perspectives regarding barriers precluding their participation in SDM. This narrative can improve understanding of the interactional failings and highlight patients’ expectations of their participation, thereby, enhancing targeted strategies towards improvement of the participation.

### Aim

In the light of the foregoing observations, the study sought to describe perspectives concerning barriers to participation in shared decision making among DM patients in Malawi.

## Methods

### Research design

The study employed the qualitative approach to understand patients’ perspectives concerning barriers precluding their participation in SDM .

### Study setting

The study was conducted at 2 tertiary health facilities and 2 secondary ones, namely Queen Elizabeth Central Hospital and Zomba Central Hospital (ZCH), and Chikwawa and Mulanje District hospitals respectively. The facilities were selected based on an understanding that they would generate diverse views about the subject matter because they were different in terms of geographical location and offered care levels. Again, we purposively chose the public sector to reflect patients’ insights from the largest healthcare provider in Malawi.

### Study participants

Through the clinic in-charges, we purposively identified 39 participants who came for their routine DM care visits who we, in turn, asked to participate in the study. In order to diversify views obtained, we further tasked the clinic in-charge to purposively select participants’ who varied in age, sex, and DM type and duration. The eligibility of the participants depended on their willingness to participate in the interviews; ability to provide informed consent; their patronage of the facility for more than 6 months, which implied that they had long enough contact with the health facility to significantly contribute to the study. We explained the purpose of the study to the potential participants; two of the them refused participation due to time constraints. We obtained informed consent for interviews and audio-recoding from all the participants who met the criteria and agreed to participate.

### Data collection

For data collection, we used in-depth interviews (IDIs) and focus group discussions (FGDs) guided by an unstructured questionnaire (supplementary file attached). There were 15 IDIs and two FGDs of 11 participants each. Two of the patients that participated in IDIs were also included in FGDs. The first author conducted all interviews in the local language within the clinic. Apart from exploring perceptions about patient centred care, participants were asked to narrate their experiences and barriers around participation in common decisions in recent care. Saturation of responses was arrived after 13 IDIs but we continued with two more IDIs to verify saturation points. After each interview and focus group discussion, main issues arising from them were noted and summarized to participants for data verification.

We conducted the study from September to December 2019.

### Data management and analysis

For purposes of this paper, we focused our analysis on patients’ perceptions and barriers to their engagement in SDM.

The data analysis process started during data collection in the field by noting and exploring further recurrent themes. Following the data collection, we transcribed the audio data verbatim and imported it into NVivo software version 11 for analysis.

We employed Content Analysis where we familiarised, coded the data and clustered emerging thematic patterns [21]. The analysts read the transcripts repetitively to familiarise themselves with the data set. Through this step we identified the perceptions and barriers to the SDM process.

We ensured credibility by triangulating individual views in IDIs with collective ones from FGDs [22]. Transferability of the results was enhanced through diversifying the patient profiles within the DM group studied [22]. Also as a way of quality check, two random transcripts were coded by the first and the third authors concurrently almost at the beginning of the coding process. Differences in coding were discussed and resolved by negotiating dominant meanings in the controversial quotes. Then we placed quotes in the rightful thematic context. We then updated the codebook and re-coded the transcripts using the new codebook. Conformability was achieved through counterchecking the codes by another individual of a different background to help mitigate the effect of researcher’s prior predispositions that might have affected the analysis [22].

## Results

Two themes were identified; the perceived value and benefits of participating in SDM, and challenges associated with patients’ engagement towards SDM which has subthemes.

### Perceived value and benefits of participating in SDM

Participants generally felt that adherence to decisions made pertaining to diabetic care would be enhanced if the patients themselves were part of the decisions made:

“ … A patient should be asked and should be allowed to give his/her suggestions, that way it would be easier for him/her to follow the decisions made [ … ]. So it is very important to inquire from us first. [ … ] But just making decisions without the knowledge of the patient sometimes becomes a problem.” (Male participant 2 rural FGD)

Further, the participants yearned for adequate dialogue and as pre-requisite meaningful engagement towards seeking clarification concerning patients’ stories.

‘However there has to be a good motivation, you [the provider] should ask questions, I should answer. I should ask questions and you should explain to me that for your problem to be over, you should do this and I should try that. “How are you feeling now?” “Everything is well.” By doing that we have helped each other questions and seeking clarifications from patients.’ (Female participant 2 FGD urban)

The excerpt further describes the patients’ expectation and perceived value of engaging them in SDM and arriving at collaborative care plans.

### Challenges associated with patients’ engagement towards SDM

The theme expresses patients’ struggles and vulnerabilities faced in trying to ask questions in pursuit to engage their providers in SDM. The hostility of the interactional atmosphere and their own perceptions towards provider-patient interactions are testified. The following subthemes emerged from the theme:

- **Fearful and dismissive ambience**

Some patients reported that they were unable to contribute to the discussion because the patient -provider interactional atmosphere was so fearful and dismissive that they retreat to a submissive and unknowledgeable role.

‘What scares us is, whenever you are talking too much and suggesting ideas to the doctor, they just ask you one question, “Are you the doctor? Just go and do whatever you are told.” And then we don’t have anything to say’. (Female participant 9, urban FGD)

The feeling of fear in patients was perpetuated by the possibility of being denied the day’s intended service if they asked questions aimed at achieving engagement. Notably, patients noticed providers’ unspoken cues around paternalistic inclinations, and their reluctance to involve them in care decisions.

‘ … when you start asking the doctor too many questions, they leave you because they assume that you know everything. It is just out of luck when they write a prescription for you and that is why we are resilient to answer back. You can even tell that this doctor is angry at me and you don’t ask any more questions. You just wait for the prescription and then off you go. We should say the truth, a patient should not be wise more than the doctor. Just listen to whatever s/he is saying even though s/he is wrong.’ (Female Participant 9, FGD urban)

Patients also perceived dismissive interactions as a deterrent to engagement towards SDM. They, reported their experiences with service providers who did not listen to patients’ ideas.

‘There are some provider [s], especially doctors, before you finish explaining, they have already written in your book so what can you do? Can you continue talking with them? Doctors are the ones who are supposed to listen to our complaints but most of them dispose us off even before we finish talking’. (Female participant IDI urban)

b. **Insufficient opportunities for dialogue**

The theme highlights sufficiency level of dialogue between the patients and the provider regarding the problem and its management to enable sound participation in SDM. With regards to alternative treatment options, patients expressed their potential to offer experiential insights into their diseases and their management, however, they confirmed that providers missed opportunities to tap into patients’ expertise through dialogue.

‘Just imagine a fresh doctor coming from College of Medicine trying to tell a patient who has been diabetic for 18 years. Who knows much there? It is us patients. That is why there is a need for that good doctor-patient relationship. Just because they learnt theory doesn’t mean that they know everything. We are the ones who know much because we are the patients and they are supposed to listen to us. They should tell us what they learnt, the new technologies coming in but we should also tell them about our disease because we have an experience of the condition and by doing that we will help each other.’ (Female participant 7, urban FGD)

Some patients with experiential knowledge, ironically, found it difficult to engage with their providers in dialogue and when they did, they felt that it would be futile for them to propose options because the opinions could not be heard.

‘It is very difficult to have an opportunity to sit down with the doctor and discuss because mostly they feel like those issues should be left with the researchers and the diet which they have given us is ideal. So I don’t think that even if we sit down and discuss, it won’t change anything.’ (Male participant, IDI urban)

### Patients’ own perceptions towards dialogue and SDM

It was noted that patients’ own perceived inappropriateness of exchanging ideas, asking questions about their care with their providers contributed to their passivity in the interaction.

‘My suggestions [are] if you are [a] patient and you are meeting the doctor, there is no way you can be exchanging ideas. The doctor just tells you that depending on your results, follow this, this and this. I will prescribe these drugs for you. I don’t think a patient can have the audacity to be telling the doctor what to do as if you know about health. You just listen and take whatever the doctor has told you. (Participant 5 urban FGD)

The data analysis revealed that patients’ own perception of inadequate health literacy deterred their engagement in meaningful dialogue with providers. Similarly, patients believed that the providers were their sole help hence their need to trust whatever they said and, to gratefully receive and comply with care packages without questioning anything.

‘I would like to comment on what number 5 [participant 5] said, that we so much put our trust in the doctors. When they tell us this is how you are going to be taking your medicine, we listen and we do not disagree because we know that the doctor knows his/her job and we don’t have a right to disagree. That is why I said even if you have been prescribed the wrong medicine, there is nothing you can do unless if there is someone who also knows about medicine’. (Female participant 8, urban FGD)

‘So whenever we come here, we take the doctors as our help. We trust whatever they say.’ (Female participant 2, urban FGD)

## Discussion

While patients seem to value and yearn for engagement in SDM in clinical encounters, the narratives highlight that active participation among patients with DM is hampered by a non-inviting interactional atmosphere, insufficiency of dialogue, and patients’ own predispositions towards SDM. The themes overlap greatly, hence the need for the results and interpretations to be considered in the context of sensitivities of interactional power imbalances between the patients and their service providers.

Our study highlights the non-inviting, fearful and power imbalanced atmosphere as a deterrent to SDM. Furthermore, the unfavourable ambience compels patients to assume passive, subservient and unknowledgeable roles during medical encounters. Especially in the African context, this finding is supported by records of chronic care encounters being largely paternalistic with power imbalances that tilt against the patient [7, 23, 24]. The interactional power imbalances are reportedly prominent where patients are socially and medically disadvantaged, among populations with low patient medical literacy and, those accessing non-fee paying services [25]. These factors have been reported to further reduce patient awareness of decisional freedom and assertiveness to initiate participation [12, 16]. Particularly, in resource-limited contexts, patients opt for passivity due to fear of risking their only opportunity to receive care upon asking questions that anger providers who perceive them as challenging their authority and trust [19]. Thus, passivity concerning patient care decisions also reflect poor patient provider relationship and patients’ way of navigating felt hostility.

The reported contradiction between patients’ desire for SDM and presumed trust towards their providers is not surprising and yet an important finding to this study. Patients’ accounts about powerlessness and futility regarding decisional control during clinical encounters are not only profound but they also partly explain the apparent contradiction. Consequently, due to futility perceived while trying to engage their providers in SDM, the patients portray their presumed trust out of deep vulnerability and helplessness by playing the role of good patient to win provider favours [19]. Meaner et al. supports this finding, arguing that it is patients’ vulnerability, feeling unsafe and destitute that push them into blindly trusting their providers or risk being denied a services or being treated badly [26, 27]. Additionally, the results in our study show that patients’ presumed trust in their providers hardly emanates from sufficient information shared or good interactional relationship in the dyad. To make SDM a reality, Joseph-Williams et al. purports that patient provider trust must be born out of positive relations to the extent that the patient and the provider should have a “shared mind” [28]. This implies the existence of enough opportunities for dialogue with optimal exchange of information and investment into relationship building over time [29]. Although having total trust in their providers can lead to patients’ passivity concerning SDM, providers in the low resourced context need to interpret patients’ trust regarding SDM with caution to clear the ambivalence.

Concerning inadequate informational exchange through dismissive and hurried interactions deterring meaningful participation in SDM, Ringdal et al. confirms that time pressed interactions hardly promote patients’ medical literacy [27]. Medical literacy does not only facilitate access, appraisal and application of information; it also fosters patients’ feeling of decisional control and responsibility for meaningful SDM [27]. Within the time constrained DM care settings of Malawi, the perception that information sharing is time consuming may seem legitimate. However, providers should consider that the additional interactional time spent on dialogue is, usually, marginal [30] yet its value in reducing unwarranted retreatments, improving self-efficacy and tilting power balances towards the patients with DM is significant [10, 31]. Therefore the cost-effectiveness of the extra time must be weighed against potential gains of setting and attaining personalised goals especially in poorly controlled diabetic patients [13, 32]. Considering that DM medical encounters are repetitive, it would ease time pressure if providers could delegate or task shift information sharing to other cadres, and consider building patients’ medical literacy as a longitudinal task over several encounters. These acts could, over time, capacitate patients along the SDM participation ladder [13, 33].

Patients’ attitudes about engagement in SDM need investigation if meaningful SDM is to be achieved in clinical encounters. It is a known fact that a patient’s past experiences, disease condition and socio-cultural inclinations can shape their perception, abilities and attitudes towards SDM [19]. Particularly, previous studies show that patients’ perceptions that providers knowledge and authority is supreme over theirs, hence non-contestable. Consequently, patients assume passivity because they think that their health knowledge, experience is less important in the dialogue [25, 29]. While we acknowledge that patients’ attitudes that are deeply engraved in socio-cultural sensitivities are hard to change, patient education strategies that addresses the drivers to the passivity and focus on patient empowerment and enablement towards SDM are urgent in Malawi’s chronic care context.

It is evident that patients with DM are likely to participate in SDM if they are solicited within a conducive environment where they genuinely feel safe and empowered to contribute to the discussion [1, 34]. While interactional time is an important contsraint in SDM, it remains the providers responsibility to create a conducive ambience for SDM [26, 27]. Unfortunately, medical training in Malawi is still largely biomedically oriented with emphasis and reward given to technical rather than interpersonal skills [23]. This has perpetuated the partenalistic tendencies among providers. Thus, to effect the paternalistic professional culture, attitudes and routines needs to change among providers through transformative medical and nursing education that fosters active participation, relinquishing power to the patient to the necessary extent [35]. These soft skills and competencies are urgently needed.

In Malawi, while some clinical specialties such as family medicine and nursing are working towards providers’ culture change, the initial step for SDM is the relinquishing of power to patients to the extent where possible. As a starting point, a review of models of delivery and accreditation of evidence-based medicine is necessary. Specifically, a review of practical ways of integrating personalised attributes to care, rapport building and participatory skills as longitudinal competencies in pre-service and in-service medical and nursing training [35]. Through this transformative medical education, to further challenge provider attitudes about interactional power dynamics and, encourage them to regard patients as active partners and acknowledging that patients possess complementary knowledge relevant to management of their disease. In order to improve on accountability concerning these important skills and attitudes, training institutions ought to make deliberate effort to accredit the stated skills and attitudes during student and staff appraisals in training and clinical practice.

In an effort to facilitate effective partnerships and safe spaces for dialogue, we advocate the exploration and use of participatory approaches to patient education that encourages provider-patients dialogue and asking questions [35]. This has potential to improve awareness and responsibility over decisional during among patients.

### Limitations of the study

The study was conducted in the diabetic population of Southern and Eastern Regions of Malawi due to financial constraints. Therefore, the perspectives that transpired in it may not be generalizable to the general patient population. However, due to the repetitiveness of the health interactions between the healthcare system and DM patients we were of the opinion that their perspectives may enrich the goals of this study.

The narratives in this paper are limited to patients’ perspectives on barriers precluding patients’ participation in SDM. Therefore, the gaps presented may not be comprehensive, falling short of comparative analysis with perspectives from their providers.

## Conclusions

The patients’ perspectives narrated in this paper have unearthed the perceived value, relevant challenges and deep vulnerabilities associated with patients’ participation in their medical decisions during clinical encounters. Even though most Malawian patient-provider encounters are time pressed, it is evident that patients yearn to be effective partners in their care. While the reasons may be numerous; from individual to contextual issues, the findings can guide the needed practical changes in culture, training and care routines that strengthen patient participation in SDM in DM care context.

## Supplementary Information

**Additional file 1.**

## Data Availability

The datasets used and/or analysed during the current study are available from the corresponding authors on reasonable request.
