# Supplementary material for: An insight into patients’ perspectives on barriers affecting participation in shared decision making among patients with diabetes mellitus in Malawi
Source: BMC Prim Care. 2022 Mar 10;23:42. doi: 10.1186/s12875-022-01635-9 (PMC8915511; doi:10.1186/s12875-022-01635-9)
Supplement: Supplementary file 1 — Additional file 1. [file 12875_2022_1635_MOESM1_ESM.docx]

**Manuscript Title:** An insight into patients’ perspectives on their participation in medical decision making in Diabetes Mellitus care in Malawi.

**Interview guide Title:** Un-structured interview guide and probes for diabetics Mellitus patients’ in-depth interviews and focus group discussion: exploring the conceptualisation of patient centred care.

**Authors:**

**Martha Makwero^1, 2^, Adamson S. Muula^2^, Felix Chima Anyanwu^1^, Jude Igumbor^1^**

^1^ School of Public Health, Faculty of Health Sciences, University of the Witwatersrand, Johannesburg, South Africa

^2^ Department of Public health, University of Malawi, College of Medicine, Blantyre 3, Malawi

- Please tell me about the care that you received at this clinic recently
  - Please explain about the kind of interaction had between you and your provider
  - Tell me about the expectations of the care you want to receive at this clinic?
  - If you were to share with somebody your experiences about the care you received at this clinic, what would you say?
- For the care to be “centred on you”, what does that entail to you? What should happen during in the interaction with your provider to make such care “centred on you”, What key elements of the interaction with your provider would make such care “centred on you?” ( including cues that point to some important elements of PCC) or (use case vignettes of some PCC dimensions)
- *As an example do you have an opportunity to sit down to find a consensus of what should be done about your condition*
  - *Please explain; how did it go?*
  - *Use of hypothetical scenarios such as: “I would like to give you a scenario, maybe you have a high blood sugar, and you have ideas on how best you can manage the condition whilst your doctor has a contrary idea how does the interaction go?*
- Please tell me about the practice of care that is “centred on you” among HCWs at this facility:
  - In your opinion, what do you think are the factors (both positive and negative) that are influencing the type of patient-provider interactions that you experience?
  - What do you think are the enabling factors for the HCWs to render care that is deemed to be “centred on you”?
  - (In case providers already do provide care centred on you) What do you think would help reinforce the interactions that you have at this facility?
- Do you have any questions?

NB: This document represents a question guide ; not only related to this manuscript
